# *Vital Signs:* Tobacco Product Use Among Middle and High School Students — United States, 2011–2018

**DOI:** 10.15585/mmwr.mm6806e1

**Published:** 2019-02-15

**Authors:** Andrea S. Gentzke, MeLisa Creamer, Karen A. Cullen, Bridget K. Ambrose, Gordon Willis, Ahmed Jamal, Brian A. King

**Affiliations:** ^1^Office on Smoking and Health, National Center for Chronic Disease Prevention and Health Promotion, CDC; ^2^Center for Tobacco Products, Food and Drug Administration, Silver Spring, Maryland; ^3^Tobacco Control Research Branch, National Cancer Institute, National Institutes of Health, Rockville, Maryland.

## Abstract

**Introduction:**

Tobacco use is the leading cause of preventable disease and death in the United States; nearly all tobacco product use begins during youth and young adulthood.

**Methods:**

CDC, the Food and Drug Administration, and the National Cancer Institute analyzed data from the 2011–2018 National Youth Tobacco Surveys to estimate tobacco product use among U.S. middle and high school students. Prevalence estimates of current (past 30-day) use of seven tobacco products were assessed; differences over time were analyzed using multivariable regression (2011–2018) or t-test (2017–2018).

**Results:**

In 2018, current use of any tobacco product was reported by 27.1% of high school students (4.04 million) and 7.2% of middle school students (840,000); electronic cigarettes (e-cigarettes) were the most commonly used product among high school (20.8%; 3.05 million) and middle school (4.9%; 570,000) students. Use of any tobacco product overall did not change significantly during 2011–2018 among either school level. During 2017–2018, current use of any tobacco product increased 38.3% (from 19.6% to 27.1%) among high school students and 28.6% (from 5.6% to 7.2%) among middle school students; e-cigarette use increased 77.8% (from 11.7% to 20.8%) among high school students and 48.5% (from 3.3% to 4.9%) among middle school students.

**Conclusions and Implications for Public Health Practice:**

A considerable increase in e-cigarette use among U.S. youths, coupled with no change in use of other tobacco products during 2017–2018, has erased recent progress in reducing overall tobacco product use among youths. The sustained implementation of comprehensive tobacco control strategies, in coordination with Food and Drug Administration regulation of tobacco products, can prevent and reduce the use of all forms of tobacco products among U.S. youths.

## Introduction

Tobacco use is the leading cause of preventable disease and death in the United States; nearly all tobacco product use begins during youth and young adulthood (*1*,*2*). Cigarette smoking among U.S. youths has steadily declined over the past 2 decades (*1*,*2*). However, recent changes to the tobacco product landscape (*3*) and the introduction of new electronic cigarette (e-cigarette) devices have shifted the types of tobacco products used by youths (*4*). Since 2014, e-cigarettes have been the most commonly used tobacco product among U.S. middle and high school students (*5*).

Although e-cigarettes have the potential to benefit adult smokers if used as a complete substitute for combustible tobacco smoking (*1*), the use of any form of tobacco product by youths is unsafe (*3*). E-cigarettes typically contain nicotine (*3*,*4*). The Surgeon General has concluded that exposure to nicotine during adolescence can cause addiction and harm the developing adolescent brain (*3*). This report provides the most recent national estimates of tobacco product use among U.S. middle and high school students.

## Methods

The National Youth Tobacco Survey (NYTS) is an annual cross-sectional, voluntary, school-based, self-administered, pencil-and-paper survey of U.S. middle school (grades 6–8) and high school (grades 9–12) students.* A three-stage cluster sampling procedure is used to generate a nationally representative sample of U.S. students attending public and private schools in grades 6–12. This report used data from eight NYTS waves (2011–2018); sample sizes (response rates) were 18,866 (72.7%) in 2011; 24,658 (73.6%) in 2012; 18,406 (67.8%) in 2013; 22,007 (73.3%) in 2014; 17,711 (63.4%) in 2015; 20,675 (71.6%) in 2016; 17,872 (68.1%) in 2017; and 20,189 (68.2%) in 2018.

Participants were asked about use of seven tobacco products: cigarettes, cigars (cigars, little cigars, and cigarillos), smokeless tobacco,^†^ e-cigarettes,^§^ hookahs,^¶^ pipe tobacco,** and bidis.^††^ Current use of each product was defined as use on ≥1 day during the past 30 days. Any tobacco product use was defined as current use of one or more of the seven assessed tobacco products. Use of ≥2 tobacco product types was defined as current use of two or more of the seven assessed tobacco products. Any combustible tobacco product use was defined as current use of one or more of the following: cigarettes, cigars, hookahs, pipe tobacco, and bidis. Among respective users, frequent tobacco product use, defined as use on ≥20 of the past 30 days, was assessed for cigarettes, cigars, smokeless tobacco, e-cigarettes, and hookahs.^§§^

Data were weighted to account for the complex survey design and adjusted for nonresponse. National prevalence estimates with 95% confidence intervals were computed; population totals were estimated from extrapolated probability weights. In 2018, current use estimates were determined for any tobacco product overall, ≥2 tobacco products, any combustible tobacco product, and individual tobacco products, overall and by selected demographics (sex and race/ethnicity) within each school level (middle and high school). The presence of linear and nonlinear (quadratic) trends during 2011–2018 were assessed, adjusting for sex, race/ethnicity, and grade level.^¶¶^ Differences in current and frequent tobacco product use during 2017–2018 were assessed by t-test. For all analyses, p-values <0.05 were considered statistically significant.

## Results

In 2018, 27.1% of high school students (an estimated 4.04 million) reported current use of any tobacco product, including 13.9% (2.07 million; 51.3% of current tobacco product users) who used any combustible tobacco product and 11.3% (1.68 million; 41.7% of current tobacco product users) who used ≥2 tobacco product types (Table). E-cigarettes were the most commonly used tobacco product among high school students (20.8%), followed by cigarettes (8.1%), cigars (7.6%), smokeless tobacco (5.9%), hookahs (4.1%), and pipe tobacco (1.1%). Use of any tobacco product, ≥2 tobacco products, e-cigarettes, cigarettes, cigars, smokeless tobacco, and pipe tobacco was higher among males than females (p<0.05). Among high school students, use of any tobacco product was reported by 32.4% of non-Hispanic whites (whites), 21.7% of Hispanics, 18.4% of non-Hispanic students of other races, and 17.4% of non-Hispanic blacks (blacks). E-cigarettes were the most commonly used tobacco product among white (26.8%) and Hispanic (14.8%) high school students; cigars were the most commonly used tobacco product among black high school students (9.2%).

**TABLE:** Estimated prevalence of tobacco product use in the past 30 days, by product,* school level, sex, and race/ethnicity^†^ — National Youth Tobacco Survey, United States, 2018

School level/Tobacco product	% (95% CI)						Total	
	Sex		Race/Ethnicity					
	Female	Male	White, non-Hispanic	Black, non-Hispanic	Hispanic	Other race, non-Hispanic	Estimated no. of users^§^	% (95% CI)
**High school students**								
Any tobacco product^¶^	24.9 (22.9–26.9)	29.1 (27.1–31.3)	32.4 (30.4–34.4)	17.4 (14.5–20.7)	21.7 (19.4–24.1)	18.4 (15.0–22.4)	**4,040,000**	**27.1 (25.3–29.0)**
Any combustible tobacco**	13.0 (11.3–15.0)	14.6 (13.3–16.0)	14.7 (13.0–16.6)	13.2 (10.8–15.9)	13.7 (11.8–15.7)	8.1 (5.8–11.1)	**2,070,000**	**13.9 (12.6–15.4)**
≥2 Tobacco products^††^	9.3 (8.0–10.9)	13.1 (11.7–14.6)	13.6 (12.1–15.4)	5.5 (4.0–7.5)	9.9 (8.4–11.5)	6.3 (4.1–9.6)	**1,680,000**	**11.3 (10.1–12.6)**
E-cigarettes	18.8 (16.7–21.1)	22.6 (20.6–24.8)	26.8 (24.7–29.0)	7.5 (5.5–10.2)	14.8 (12.9–17.0)	14.5 (10.8–19.1)	**3,050,000**	**20.8 (18.8–22.9)**
Cigarettes	7.3 (6.1–8.7)	8.8 (7.6–10.2)	9.9 (8.5–11.6)	3.2 (2.3–4.6)	7.2 (5.8–8.8)	4.4 (2.5–7.6)	**1,180,000**	**8.1 (7.1–9.3)**
Cigars	6.0 (4.9–7.4)	9.0 (8.1–10.0)	7.8 (6.7–9.1)	9.2 (6.8–12.4)	7.3 (5.9–9.1)	3.4 (2.0–5.7)	**1,100,000**	**7.6 (6.7–8.6)**
Smokeless tobacco	3.3 (2.7–4.0)	8.4 (6.9–10.1)	7.6 (6.2–9.2)	2.2 (1.4–3.3)	4.2 (3.3–5.4)	3.0 (1.7–5.3)	**870,000**	**5.9 (5.0–7.0)**
Hookahs	4.1 (3.2–5.3)	4.0 (3.4–4.8)	3.3 (2.6–4.1)	3.7 (2.7–5.2)	6.0 (4.7–7.7)	4.1 (2.8–6.1)	**590,000**	**4.1 (3.5–4.9)**
Pipe tobacco	0.8 (0.6–1.2)	1.4 (1.1–1.8)	1.1 (0.8–1.6)	—^§§^	1.4 (0.9–2.1)	—	**160,000**	**1.1 (0.9–1.4)**
**Middle school students**								
Any tobacco product^¶^	6.3 (5.4–7.4)	8.0 (6.9–9.3)	6.6 (5.5–7.8)	6.8 (5.2–9.0)	9.5 (8.0–11.2)	3.8 (2.1–6.6)	**840,000**	**7.2 (6.3–8.1)**
Any combustible tobacco**	2.9 (2.2–3.7)	3.7 (2.9–4.6)	2.5 (1.7–3.4)	4.4 (3.0–6.3)	4.7 (3.9–5.7)	—	**380,000**	**3.3 (2.7–4.0)**
≥2 Tobacco products^††^	1.9 (1.4–2.5)	2.8 (2.2–3.5)	2.1 (1.5–3.0)	1.5 (0.8–2.7)	3.6 (2.9–4.4)	—	**270,000**	**2.4 (1.9–2.9)**
E-cigarettes	4.8 (3.9–5.7)	5.1 (4.2–6.2)	4.9 (4.0–5.9)	3.0 (2.1–4.2)	6.6 (5.1–8.5)	—	**570,000**	**4.9 (4.2–5.8)**
Cigarettes	1.5 (1.1–2.0)	2.1 (1.6–2.7)	1.6 (1.1–2.4)	—	2.4 (1.8–3.1)	—	**200,000**	**1.8 (1.4–2.2)**
Cigars	1.6 (1.2–2.1)	1.7 (1.3–2.3)	1.1 (0.7–1.6)	2.9 (1.8–4.5)	2.2 (1.6–2.9)	—	**190,000**	**1.6 (1.3–2.1)**
Smokeless tobacco	0.9 (0.6–1.3)	2.7 (2.1–3.6)	1.8 (1.3–2.6)	—	2.2 (1.7–3.0)	—	**210,000**	**1.8 (1.5–2.3)**
Hookahs	1.0 (0.7–1.4)	1.5 (1.0–2.1)	0.8 (0.5–1.3)	—	2.2 (1.6–3.0)	—	**140,000**	**1.2 (0.9–1.6)**
Pipe tobacco	0.4 (0.2–0.6)	0.3 (0.2–0.5)	—	—	0.6 (0.4–1.0)	—	**30,000**	**0.3 (0.2–0.5)**

**Abbreviations:** CI = confidence interval; e-cigarettes = electronic cigarettes.

* Past 30-day use of e-cigarettes was determined by asking, “During the past 30 days, on how many days did you use e-cigarettes?” Past 30-day use of cigarettes was determined by asking, “During the past 30 days, on how many days did you smoke cigarettes?” Past 30-day use of cigars was determined by asking, “During the past 30 days, on how many days did you smoke cigars, cigarillos, or little cigars?” Past 30-day use of hookah was determined by asking, “During the past 30 days, on how many days did you smoke tobacco in a hookah or waterpipe?” Smokeless tobacco was defined as use of chewing tobacco, snuff, dip, snus, and/or dissolvable tobacco products. Past 30-day use of smokeless tobacco was determined by asking the following question for use of chewing tobacco, snuff, and dip: “During the past 30 days, on how many days did you use chewing tobacco, snuff, or dip?,” and the following question for use of snus and dissolvable tobacco products: “In the past 30 days, which of the following products did you use on at least one day?” Responses from these questions were combined to derive overall smokeless tobacco use. Past 30-day use of pipe tobacco (not hookahs) was determined by asking, “In the past 30 days, which of the following products have you used on at least one day?”

^†^ Blacks, whites, and others are non-Hispanic; Hispanic persons could be of any race.

^§^ Estimated total number of users was rounded down to the nearest 10,000 persons.

^¶^ Any tobacco product use was defined as use of any tobacco product (e-cigarettes, cigarettes, cigars, smokeless tobacco, hookahs, pipe tobacco, and/or bidis) on ≥1 day in the past 30 days.

** Any combustible tobacco product use was defined as use of cigarettes, cigars, hookahs, pipe tobacco, and/or bidis on ≥1 day in the past 30 days.

^††^ ≥2 tobacco products use was defined as use of ≥2 tobacco products (e-cigarettes, cigarettes, cigars, smokeless tobacco, hookahs, pipe tobacco, and/or bidis) on ≥1 day in the past 30 days.

^§§^ Dashes indicate that data are statistically unreliable because samples size was <50 or relative standard error was >0.3.

In 2018, 7.2% (an estimated 840,000) of middle school students reported current use of any tobacco product, including 3.3% (380,000; 45.8% of current tobacco product users) who used any combustible tobacco product and 2.4% (270,000; 33.3% of current tobacco product users) who used ≥2 tobacco products (Table). Among middle school students, the most commonly used tobacco produce was e-cigarettes (4.9%), followed by cigarettes (1.8%), smokeless tobacco (1.8%), cigars (1.6%), hookahs (1.2%), and pipe tobacco (0.3%). Use of smokeless tobacco, any tobacco product, and ≥2 tobacco products was higher among males than females (p<0.05). Among middle school students, use of any tobacco product was reported by 9.5% of Hispanics, 6.8% of blacks, 6.6% of whites, and 3.8% of non-Hispanic students of other races. E-cigarettes were the most commonly used tobacco product among Hispanic (6.6%), white (4.9%), and black (3.0%) middle school students.

**FIGURE 1:**
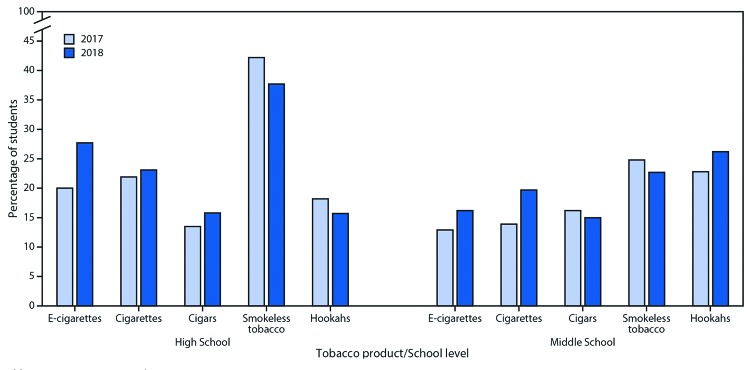
Frequent use* of selected tobacco products† among U.S. middle and high school students who currently used each tobacco product^§^ — National Youth Tobacco Survey, 2017–2018^¶^ **Abbreviation:** e-cigarettes = electronic cigarettes. * Frequent tobacco product use defined as use of each respective tobacco product on ≥20 of the past 30 days. ^†^ Frequency of use during the past 30 days was not available for pipe tobacco in the 2017 or 2018 surveys. ^§^ Among youths who currently report using each respective tobacco product, defined as a response other than "0 days" to each of the following questions: *E-cigarettes:* "During the past 30 days, on how many days did you use e-cigarettes?"; *Cigarettes:* "During the past 30 days, on how many days did you smoke cigarettes?"; *Cigars:* "During the past 30 days, on how many days did you smoke cigars, cigarillos, or little cigars?"; *Smokeless tobacco:* “During the past 30 days, on how many days did you use chewing tobacco, snuff, or dip?"; *Hookahs:* "During the past 30 days, on how many days did you smoke tobacco in a hookah or waterpipe?" For all questions, answer choices included, “0 days, 1 or 2 days, 3 to 5 days, 6 to 9 days, 10 to 19 days, 20 to 29 days, and All 30 days." ^¶^ During 2017–2018, a significant increase in frequent use of e-cigarettes was observed only among high school students (p<0.05). No significant changes were observed for any other tobacco product during 2017–2018 among middle or high school students

In 2018, frequent use among current product users in high school was 37.7% for smokeless tobacco, 27.7% for e-cigarettes, 23.1% for cigarettes, 15.8% for cigars, and 15.7% for hookahs (Figure 1). During 2017–2018, frequent e-cigarette use increased significantly by 38.5% among current e-cigarette users (from 20.0% to 27.7%); no significant change in frequent use was observed for other tobacco products. Among middle school students, frequent use among current product users was 26.2% for hookahs, 22.7% for smokeless tobacco, 19.7% for cigarettes, 16.2% for e-cigarettes, and 15.0% for cigars in 2018; no significant change in frequent use was observed for any product during 2017–2018.

Among current users of any tobacco product in 2018, exclusive use of e-cigarettes was reported by 42.0% of high school students and 42.7% of middle school students. However, among high school students who reported currently using ≥2 tobacco products, the most common combinations reported were “e-cigarettes + cigarettes” (14.8%); “e-cigarettes + cigars” (13.3%); and “e-cigarettes + smokeless tobacco” (9.0%). Among middle school students who reported currently using ≥2 tobacco products, the most common combinations reported were “e-cigarettes + cigarettes” (14.4%); “e-cigarettes + cigars” (9.1%); and “cigarettes + e-cigarettes + cigars + smokeless tobacco + hookah” (8.8%).

**FIGURE 2:**
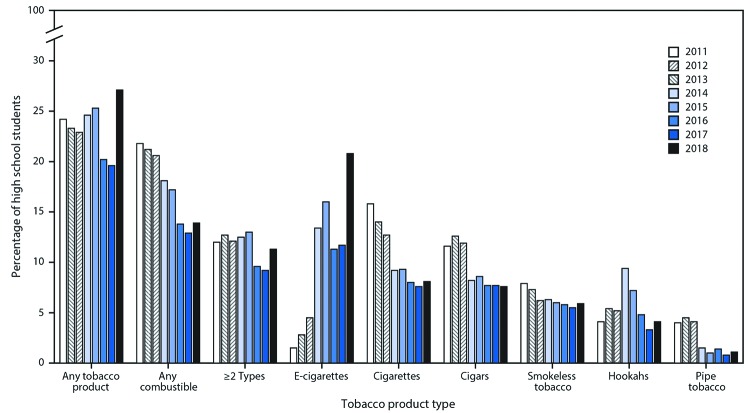
Estimated percentage of high school students who currently use any tobacco product,* any combustible tobacco product,**^†^** ≥2 tobacco product types,**^§^** and selected tobacco products — National Youth Tobacco Survey, 2011–2018**^¶^**^,^**^,^**^††^** **Abbreviation:** e-cigarettes = electronic cigarettes. * Any tobacco product use was defined as use of e-cigarettes, cigarettes, cigars, hookahs, smokeless tobacco, pipe tobacco and/or bidis (small brown cigarettes wrapped in a leaf) on ≥1 day in the past 30 days. ^†^ Any combustible tobacco product use was defined as use of cigarettes, cigars, hookahs, pipe tobacco, and/or bidis on ≥1 day in the past 30 days. ^§^ Use of ≥2 tobacco product types was defined as use of ≥2 of the following tobacco products: e-cigarettes, cigarettes, cigars, hookahs, smokeless tobacco, pipe tobacco, and/or bidis on ≥1 day in the past 30 days. ^¶^ During 2017–2018, current use of any tobacco product, ≥2 types of tobacco products, and e-cigarettes significantly increased (p<0.05). ** During 2011–2018, current use of combustible tobacco products, ≥2 types of tobacco products, cigars, smokeless tobacco, and pipe tobacco exhibited linear decreases (p<0.05). Current use of cigarettes exhibited a nonlinear decrease (p<0.05). Current use of hookahs exhibited a nonlinear change (p<0.05). Current use of e-cigarettes exhibited a nonlinear increase (p<0.05). No significant trend in use of any tobacco product overall was observed. ^††^ Beginning in 2015, the definition of smokeless tobacco included chewing tobacco/snuff/dip, snus, and dissolvable tobacco to better reflect this class of tobacco products. Thus, estimates for individual smokeless tobacco products (chewing tobacco/snuff/dip, snus, and dissolvable tobacco) are not reported. This definition was applied across all years (2011–2018) for comparability purposes.

Among high school students, during 2011–2018, no significant trend in the reported use of any tobacco product overall was observed (Figure 2). However, changes were observed for individual tobacco products over this period. A significant nonlinear increase in current e-cigarette use occurred from 2011 (1.5%) to 2018 (20.8%). During 2011–2018, significant linear declines in combustible tobacco product use (from 21.8% to 13.9%) and ≥2 tobacco product use (from 12.0% to 11.3%) occurred; by product type, significant linear declines occurred for cigars (from 11.6% to 7.6%), smokeless tobacco (from 7.9% to 5.9%), and pipe tobacco (from 4.0% to 1.1%). A significant nonlinear decline was observed for cigarettes (from 15.8% to 8.1%). A significant nonlinear change during 2011–2018 was observed for hookahs (from 4.1% to 4.1%).

**FIGURE 3:**
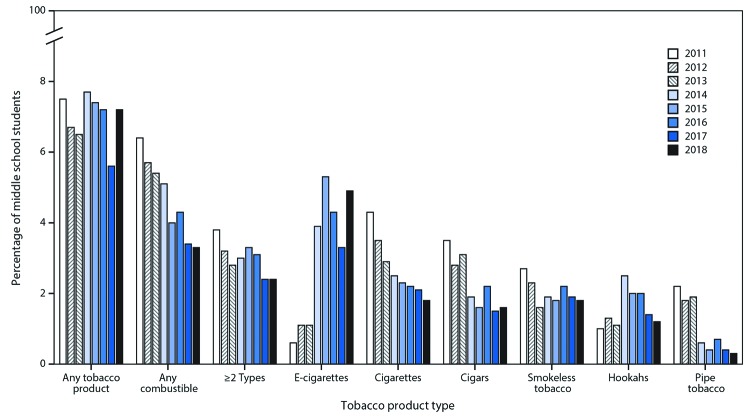
Estimated percentage of middle school students who currently use any tobacco product,* any combustible tobacco product,**^†^** ≥2 tobacco product types,**^§^** and selected tobacco products — National Youth Tobacco Survey, 2011–2018**^¶^**^,^**^,^**^††^** **Abbreviation:** e-cigarettes = electronic cigarettes. * Any tobacco product use was defined as use of e-cigarettes, cigarettes, cigars, hookahs, smokeless tobacco, pipe tobacco and/or bidis (small brown cigarettes wrapped in a leaf) ≥1 day in the past 30 days. ^†^ Any combustible tobacco product use was defined as use of cigarettes, cigars, hookahs, pipe tobacco, and/or bidis on ≥1 day in the past 30 days. ^§^ Use of ≥2 tobacco product types was defined as use of ≥2 of the following tobacco products: e-cigarettes, cigarettes, cigars, hookahs, smokeless tobacco, pipe tobacco, and/or bidis on ≥1 day in the past 30 days. ^¶^ During 2017–2018, current use of any tobacco product and e-cigarettes significantly increased (p<0.05). ** During 2011–2018, current use of combustible tobacco products, ≥2 tobacco products, cigarettes, cigars, smokeless tobacco, and pipe tobacco exhibited significant linear decreases (p<0.05). Use of e-cigarettes exhibited a significant nonlinear increase (p<0.05), and use of hookahs exhibited a nonlinear change (p<0.05). No significant trend in use of any tobacco product overall was observed. ^††^ Beginning in 2015, the definition of smokeless tobacco included chewing tobacco/snuff/dip, snus, and dissolvable tobacco to better reflect this class of tobacco products. Thus, estimates for individual smokeless tobacco products (chewing tobacco/snuff/dip, snus, and dissolvable tobacco) are not reported. This definition was applied across all years (2011–2018) for comparability purposes.

Among middle school students, no significant change in use of any tobacco product overall occurred during 2011–2018 (Figure 3). However, changes for individual tobacco products were observed. A significant nonlinear increase in e-cigarette use occurred (from 0.6% to 4.9%) during 2011–2018. A significant linear decline was observed for combustible tobacco product use (from 6.4% to 3.3%), ≥2 tobacco products use (from 3.8% to 2.4%), cigarettes (from 4.3% to 1.8%), cigars (from 3.5% to 1.6%), smokeless tobacco (from 2.7% to 1.8%), and pipe tobacco (from 2.2% to 0.3%); a significant nonlinear change occurred for hookah smoking (from 1.0% to 1.2%).

During 2017–2018, use of any tobacco product increased significantly by 38.3% (from 19.6% to 27.1%) among high school students (Figure 2) and by 28.6% (from 5.6% to 7.2%) among middle school students (Figure 3). Current use of ≥2 tobacco products increased significantly by 22.8% (from 9.2% to 11.3%) among high school students. Current e-cigarette use increased significantly by 77.8% (from 11.7% to 20.8%) among high school students and by 48.5% (from 3.3% to 4.9%) among middle school students during 2017–2018; no significant changes in use of other tobacco products was observed during this period, irrespective of grade level.

## Conclusions and Comment

In 2018, approximately one in four U.S. high school students and one in 14 middle school students reported current use of any tobacco product. Among both high school and middle school students, current use of e-cigarettes increased considerably between 2017 and 2018, reaching epidemic proportions, according to the U.S. Surgeon General (*4*); approximately 1.5 million more youths currently used e-cigarettes in 2018 (3.6 million) compared with 2017 (2.1 million) (*5*). However, no significant change in current use of combustible tobacco products, such as cigarettes and cigars, was observed in recent years (*5*) or during 2017–2018. This indicates that e-cigarettes were the driver of the observed increase in any tobacco product use. The recent changes in patterns of use of e-cigarettes and other tobacco products during 2017–2018 erased the decline in any tobacco product use that occurred in previous years (*5*).

E-cigarettes have been the most commonly used tobacco product among U.S. youths since 2014 (*5*). Before 2018, the prevalence of e-cigarette use by U.S. high school students had peaked in 2015 before declining by 29% during 2015–2016 (from 16% to 11.3%) (*6*); this decline was the first ever recorded for e-cigarette use among youths in the NYTS since monitoring began, and it was subsequently sustained during 2016–2017 (*5*). However, current e-cigarette use increased by 77.8% among high school students and 48.5% among middle school students during 2017–2018, erasing the progress in reducing e-cigarette use, as well as any tobacco product use, that had occurred in prior years (*7*).

This recent increase in e-cigarette use among youths is consistent with observed increases in sales of the e-cigarette JUUL (*8*), a USB-shaped e-cigarette device with a high nicotine content that can be used discreetly and is available in flavors that can appeal to youths. A single prefilled liquid nicotine JUUL pod contains as much nicotine as a pack of cigarettes (*9*). Media reports and a survey indicate that JUUL devices are being used among youths in schools, including inside bathrooms and classrooms.*** JUUL entered the U.S. market in 2015 and subsequently became a commonly used tobacco product among U.S. youths (*10*). Sales of JUUL increased by approximately 600% during 2016–2017 (*8*) and increased even further through 2018 (*10*). By December 2017, JUUL held the largest market share of any e-cigarette (*8*). Thus, given that NYTS is fielded annually in the spring, the 2018 data are the first to reflect the impact of rising sales of JUUL and other USB-shaped devices on e-cigarette and overall tobacco product use among U.S. youths.

Any form of tobacco product use among youths, irrespective of frequency, is unsafe (*1*–*4*). During 2017–2018, frequent e-cigarette use increased significantly by 38.5% among high school student users. Thus, in addition to more youths using e-cigarettes overall, current e-cigarette users also are using them more frequently.

Furthermore, among current tobacco product users, approximately 40% of high school students and one third of middle school students reported currently using more than one tobacco product; the prevalence of using two or more tobacco products increased significantly by 22.8% among high school students during 2017–2018. E-cigarettes were the most commonly reported product used in combination with other products among both middle and high school students in 2018. Most e-cigarettes contain nicotine (*11*), which is highly addictive and can harm the developing adolescent brain (*3*). Among youths, symptoms of nicotine dependence are increased in multiple tobacco product users than in single product users (*12*). In addition, some evidence suggests that e-cigarette use increases the risk for ever using cigarettes among youths, and that e-cigarette use might increase the frequency and intensity of subsequent cigarette smoking (*13*).

Differences in individual tobacco product use were also observed across population groups. In 2018, e-cigarettes were the most commonly used product among all racial/ethnic groups except black high school students, among whom cigars were the most commonly reported product. Targeted advertising of cigars in locations with a greater proportion of black residents, a relatively lower price, and the availability of cigars for purchase as a single unit might contribute to higher cigar smoking among blacks (*14*).

The findings in this report are subject to at least three limitations. First, changes in the wording and placement of survey questions for certain tobacco products during 2011–2018 might limit comparability of estimates between years. Second, data were self-reported and might be subject to recall and response bias. Finally, findings might not be generalizable to all youths, including those who are home-schooled, have dropped out of school, or are enrolled in alternative schools. However, in 2016, nearly 97% of students aged 10–17 years were enrolled in school.^†††^

Several factors continue to promote and influence tobacco product use among youths, including exposure to tobacco product advertising and imagery through various media, as well as the availability of flavored tobacco products (*2*,*3*,*15*,*16*). The sustained and comprehensive implementation of population-based strategies, in coordination with the regulation of tobacco products by the Food and Drug Administration (*17*), and continued research investments and cessation-related initiatives, including Smokefree Teen by the National Institutes of Health’s National Cancer Institute^§§§^ can reduce all forms of tobacco product use and initiation among U.S. youths (*1*–*3*). As a direct result of the considerable increase in e-cigarette use among youths during 2017–2018 (*7*), in November 2018, the Food and Drug Administration announced several proposed new steps to protect youths, including restricting sales of flavored e-cigarettes (other than tobacco, menthol, mint, or nonflavored) to physical locations with age restrictions or online with heightened age verification procedures, and plans to advance notices of proposed rulemaking that would ban menthol cigarettes and cigars and all other flavored cigars (*18*). Additional strategies to reduce tobacco product use among youths include increasing the price of tobacco products, implementing comprehensive smoke-free policies, implementing advertising and promotion restrictions and national antitobacco public education media campaigns, and implementing and enforcing policies that raise the minimum age of purchase for tobacco products to 21 years (*1*,*3*,*19*,*20*).

**Summary**

**What is already known about this topic?**

Tobacco use is the leading cause of preventable disease and death in the United States; nearly all tobacco product use begins during youth and young adulthood.

**What is added by this report?**

In 2018, 4.04 million high school students and 840,000 middle school students currently used any tobacco product; e-cigarettes were the most commonly used product. Driven by an increase in e-cigarette use, current tobacco product use significantly increased among high school and middle school students during 2017–2018, erasing the decline in tobacco product use among youths that occurred in previous years.

**What are the implications for public health practice?**

Sustained implementation of proven population-based strategies, in coordination with Food and Drug Administration regulation of tobacco products, is important for reducing tobacco product use and initiation among U.S. youths.

## Footnotes

* https://www.cdc.gov/tobacco/data_statistics/surveys/nyts/index.htm.

† Beginning in 2015, the definition of smokeless tobacco included chewing tobacco/snuff/dip, snus, and dissolvable tobacco to reflect this class of tobacco products better. Thus, estimates for individual smokeless tobacco products (chewing tobacco/snuff/dip, snus, and dissolvable tobacco) are not reported.

§ During 2011–2013, e-cigarette use was assessed by the question “In the past 30 days, which of the following products have you used on at least one day?” and the response option, “Electronic cigarettes or e-cigarettes such as Ruyan or NJOY.” In 2014, current use of e-cigarettes was assessed by the question “During the past 30 days, on how many days did you use e-cigarettes such as Blu, 21st Century Smoke, or NJOY?” During 2015–2018, e-cigarette questions were preceded by an introductory paragraph defining the product. In 2015, current use of e-cigarettes was assessed by the question “During the past 30 days, on how many days did you use electronic cigarettes or e-cigarettes?” During 2016–2018, current use of e-cigarettes was assessed by the question “During the past 30 days, on how many days did you use e-cigarettes?”

¶ During 2011–2015, current hookah smoking was assessed by the question “In the past 30 days, which of the following products have you used on at least one day?” Hookah was the fourth or fifth response option during 2011–2013, the first option in 2014, and the fourth option in 2015. During 2016–2018, hookah questions were preceded by an introductory paragraph defining the product; current hookah smoking was assessed by the question “In the past 30 days, on how many days did you smoke tobacco in a hookah or waterpipe?”

** During 2011–2013, pipe tobacco use was assessed by the question “During the past 30 days, on how many days did you smoke tobacco in a pipe?” During 2014–2018, current use of pipe tobacco was assessed by the question “In the past 30 days, which of the following products have you used on at least one day?” and the response option “Pipes filled with tobacco (not waterpipe).” Pipe tobacco was the second response option available in 2014, the fifth option in 2015, and the second option during 2016–2018.

†† In 2018, bidis was assessed by the question, “In the past 30 days, which of the following tobacco products have you used on at least one day?” and the response option, “Bidis (small brown cigarettes wrapped in a leaf).” Beginning in 2018, prevalence estimates are not provided for bidis by school level, sex, or race/ethnicity. However, use of bidis is captured in the composite measures of any tobacco product use, ≥2 tobacco products use, and use of combustible tobacco products to maintain consistent definitions over time.

§§ Frequency of use data were available during 2011–2018 for cigarettes, cigars, and smokeless tobacco products (chewing tobacco, snuff, dip, only). Frequency of use data were available only for certain years for e-cigarettes (2014–2018), hookahs (2016–2018), and pipe tobacco (2011–2013). Frequency of use data were unavailable for bidis, snus, and dissolvable tobacco products during 2011–2018.

¶¶ Trends were assessed using multivariable-adjusted regression analysis. A test for linear trend was significant if an overall statistically significant decrease or increase occurred during the study period. Data also were assessed for the presence of nonlinear (quadratic) trends. A significant nonlinear trend indicated that the rate of change accelerated or decelerated across the study period.

*** https://www.cdc.gov/tobacco/infographics/youth/pdfs/e-cigarettes-usb-flash-508.pdf; https://truthinitiative.org/news/nearly-1-5-youth-say-they-have-seen-juul-used-school.

††† https://www.census.gov/data/tables/2016/demo/school-enrollment/2016-cps.html.

§§§ The National Cancer Institute created Smokefree.gov to help smokers quit smoking. Smokefree.gov is a part of an effort by the U.S. Department of Health and Human Services to reduce smoking rates in the United States, particularly among certain populations. Smokefree Teen (https://teen.smokefree.gov/) is part of the Smokefree.gov initiative, with the goal to reduce the number of youths who use tobacco.
